# Influenza vaccination effectiveness for people aged under 65 years in Japan, 2013/2014 season: application of a doubly robust method to a large-scale, real-world dataset

**DOI:** 10.1186/s12879-019-4186-x

**Published:** 2019-07-05

**Authors:** Natsumi Shibata, Shinya Kimura, Takahiro Hoshino, Hisashi Urushihara

**Affiliations:** 1 Department of Drug Development and Regulatory Science, Faculty of Pharmacy, Keio University, 1-5-30 Shibakoen, Minato-ku, Tokyo, 105-8512 Japan; 2 Japan Medical Data Center Co., Ltd, Sumitomo Shibadaimon Building 12F, 2-5-5 Shibadaimon, Minato-ku, Tokyo, 105-0012 Japan; 3 Department of Economics, Faculty of Economics, Keio University, 2-15-45 Mita, Minato-ku, Tokyo, 108-8345 Japan

**Keywords:** Influenza vaccines, Vaccine effectiveness, Propensity score, Doubly robust method, Japan

## Abstract

**Background:**

Influenza vaccination is recognized as a primary public health intervention which prevents the illness of patients and relieves the societal burdens of influenza for medical community as well as the economy. To date, no effectiveness study of influenza vaccination has been conducted including a large population with a wide age span, in Japan. Here, we evaluated the clinical effectiveness of influenza vaccination in a large Japanese population.

**Methods:**

We conducted a cohort study using a large-scale claims database for employee health care insurance plans. Vaccination status was identified using plan records for influenza vaccination subsidies. We excluded people aged 65 years or more because of the unavailability of vaccination records. Effectiveness of vaccination in preventing influenza and its complication was evaluated with doubly robust methods using inversed probability treatment weighting to adjust health conscious behaviours and other confounders.

**Results:**

During the 2013/2014 influenza season, 369,425 subjects with age range from 1 to 64 years were eligible. Vaccination rate was 39.5% and an estimated odds ratio (OR) for influenza onset was 0.775 after doubly robust adjustment. Age-stratified ORs were significantly reduced in all age groups; lowest in subjects aged 1 to 4 years (0.600) and highest in those aged 13 to 19 (0.938). ORs for all the influenza complication outcomes were also statistically significant (0.403–0.709).

**Conclusions:**

We confirmed the clinical effectiveness of influenza vaccination in people aged 1 to 64 years. Influenza vaccination significantly prevented influenza onset and was more effective in reducing secondary risks of influenza complications.

## Background

The heavy burden of influenza impacts not only patients – especially children, the elderly and those at high risk – such as in lives lost, but also medical institutions and the economy, in terms of lost productivity [1, 2]. Influenza vaccination is recognized worldwide as a primary public health intervention which reduces the healthcare, economic and social burden of influenza. Group influenza vaccination would protect the whole society from influenza and its aggravation [3, 4]. In Japan, a number of private employee health insurance plans and municipalities subsidize influenza vaccination.

Against this background, no large-scale effectiveness study of influenza vaccination covering a broad spectrum of generations has yet been reported in Japan. We previously conducted a vaccine effectiveness study in the largest pediatric population to date using a claims database in Japan [5]. In the present study we used the same database to extend the study population to cover generations, from children to workers.

In this study, we investigated the clinical effectiveness of influenza vaccination in a large population in Japan using a large-scale claims database.

## Methods

### Study design

The study was conducted under a retrospective cohort design using the same claims database and analytical methodology as in our previous pediatric study of influenza vaccination[5]. The claims database was provided by Japan Medical Data Center Co., Ltd. (Tokyo, Japan) and included more than 3 million enrollees of employee health care insurance plans, run mainly by large-scale private enterprises [6]. Deidentified claims data of employees and their dependents including diagnoses, procedures, and pharmacy prescriptions were collected from the health insurance plans. Study duration was the epidemic period, starting from 1 October 2013 to 31 May 2014 [7].

Ethical approval for the study protocol and waiver for informed consent to participate were provided by the Keio University Faculty of Pharmacy Ethics Committee for Research Involving Humans (No. 160118–1), in accordance with local ethical guidance for medical research involving human subjects.

### Influenza vaccination

Seasonal trivalent inactivated and nonadjuvanted influenza vaccination is available for children aged 6 months or older and adults in Japan. The recommended dosage per season is two doses of 0.25 ml for those aged 6–24 months, two doses of 0.5 ml for those aged 3–12 years, and one dose of 0.5 ml for those over 12 years. Vaccines for the 2013/2014 season contained two influenza A strains (A/California/7/2009(X-179A)pdm09, A/Texas/50/2012(X-223)) and one influenza B strain (B/Massachusetts/2/2012(BX-51B)). Circulating viral subtypes of influenza in the 2013/2014 season were A/H1N1pdm09 (43%), A/H3N2 (21%), B/Yamagata (24%), B/Victoria (9%), and B/untyped (4%) [8].

The influenza vaccination period in Japan starts at the beginning of October and the vaccination schedule is managed by the Ministry of Health, Labour and Welfare [5]. The Japanese National insurance scheme, which provides basic universal coverage of all medical costs for all nationals, does not cover influenza vaccine. Japanese nationals have to pay the cost of vaccination out-of-pocket, except for the elderly aged 65 years or older, whose vaccination is provided at local government cost by public health service centers. Children and other adults receive influenza vaccination at community clinics or hospitals at their own cost. Several employment-based health insurance plans operated by private companies subsidize influenza vaccination and the scope of subsidy differs by the age group covered and fee. Accordingly, vaccination status and dates were identified from records in the health plans for the respective influenza vaccination subsidies.

### Study population

Subjects were employees and their dependents aged 1 to 64 years as of October 1st 2013. Enrollees were eligible if their health plan provided subsidies for influenza vaccination. Because the Japan Pediatric Society does not recommend influenza vaccination for infants aged less than 1 year, potentially biasing pediatrician preferences for who should be vaccinated in this age group [9], we excluded infants aged ≤1 year from the evaluation of vaccine effectiveness. Some health plans do not provide a subsidiary program for influenza vaccination for particular age groups, such as adults aged 20 years or older. We therefore excluded subjects who were not eligible for subsidies or whose enrollment in the health plan started on or after October 2012 or ended before May 2014, to eliminate potential misclassification of vaccination status during the 2012/2013 and 2013/2014 seasons. Because seniors aged over 64 years were strongly recommended to receive vaccination and were often supported by subsidies from local governments rather than by health insurance plans, we also excluded subjects aged over 64 years to avoid misclassification of influenza vaccination status. We further excluded patients with prolonged hospitalization, on the basis that the extended period they spent in a managed environment may have affected their probability of being vaccinated or their risk of virus exposure. Patients with prolonged hospitalization were identified as those with a record of hospitalization for ≥24 days/month over ≥7 months.

The risk period for outcome events in vaccinees was from 14 days after the date of vaccination to 31 May 2014. Subjects who experienced an outcome event within 13 days after vaccination were excluded to ensure that the effects of vaccination were accurately assessed [10]. For non-vaccinees, the whole study period was the risk period for effectiveness. Subjects who got vaccinated after having experienced an outcome event were classified as non-vaccinees and censored at the time of outcome diagnosis.

### Outcome definition

We used the same definitions for the incidences of influenza diagnosis and complications as the ones used for the preceding pediatric study, which were based on the International Statistical Classification of Diseases and Related Health Problems 10th Revision (ICD-10) codes [5]. A primary diagnosis of influenza was based on the ICD-10 codes J101, J110, J111, and J118. To test the robustness of the definition based on these codes in sensitivity analyses, three different definitions of influenza incidence were developed, as follows: 1) combination of the ICD-10 codes defined above with records of the use of a rapid-testing kit, identified by claim records for the influenza virus antigen (high-sensitive) test; 2) combination of these ICD-10 codes with a prescription for antiviral drugs, as determined using the Anatomical Therapeutic Chemical (ATC) J05B4 classification of the European Pharmaceutical Marketing Research Association; or 3) the combination of the J101 code “influenza due to identified seasonal influenza virus” and use of a rapid-testing kit. Secondary outcomes of influenza complications included pneumonia (J12-J18) and respiratory tract diseases (RTD: J00-J22, apart from the above influenza codes). There were both defined using ICD-10 codes only. Cases requiring hospitalization were additionally defined for secondary outcomes as subjects hospitalized within 3 days before or after the date of influenza diagnosis (hospitalization with influenza); subjects hospitalized within 7 days of the diagnosis date of RTD (hospitalization with RTD); and emergency hospitalization with influenza or pneumonia. We identified emergency hospitalization using claim codes of the national health insurance medical fee schedule for emergency hospitalization (A205 or A300) [11].

### Confounding factors

Covariates considered for inclusion in adjustment of potential confounders in evaluating the effectiveness of vaccination included influenza vaccination status during the prior season (2012/2013 influenza season), age at October 1 2013, employment status, gender, number of other dependents aged 0 to 15 years covered under the same insurance number (number of children aged 0 to 15 in a family), preceding onset of influenza in family members during the current influenza season, history of high-risk medical conditions, emergency hospitalization, and number of outpatient visits during or outside office hours in the prior influenza off-season (June to September). High-risk medical conditions were defined using the definition of the US Centers for Disease Control and Prevention (CDC) [12]. Family members could be identified because employees and their dependents shared the same insurance number. “Preceding onset of influenza in family members” was considered to be the risk of second infection that subjects were exposed to when a family member received any influenza diagnosis code before his/her first influenza. This was considered in both the primary analysis and also in the secondary analysis for hospitalization using influenza as outcome.

### Statistical analysis

Subject characteristics in the four age groups (1–4 years, 5–12 years, 13–19 years and 20–64 years) were summarized with descriptive statistics. Between-group comparisons were tested with the Mann-Whitney test for continuous variables and the chi-square test for categorical variables.

The primary analysis was to estimate the effectiveness of influenza vaccination in preventing the onset of influenza. The secondary analysis aimed to estimate the effect of influenza vaccination on influenza complications outcomes. First, we calculated odds ratios (ORs) of outcome events for influenza vaccination in the 2013/2014 season and other covariates using conventional multivariate logistic regression. To adjust confounding related to influenza vaccination for whole subjects and respective age groups, we next used a doubly robust method (DR) which combines a logistic regression model with inverse probability treatment weighting (IPTW) by propensity score (PS) to calculate the OR_DR_s [13]. PSs were calculated as those for the probability of being vaccinated in the 2013/2014 season by considering the covariates mentioned above and the presence of children aged 0 to 15 years in a family, but not considering preceding onset of influenza in family members or the number of children aged 0 to 15 years in a family. In the analysis for whole age subjects, age effect was modeled by linear tail-restricted cubic spline functions with 5 knots, based on percentiles on the basis that treating age effect as a linearity of age effect could not be assumed [14]. Vaccinees having a PS < 0.1 as well as non-vaccinees with PS > 0.9 were excluded because these subjects with opposite, extreme PS values were reported to have the potential to cause bias in the IPTW estimates by being excessively weighted with reciprocals of PSs for vaccinees and of (1-PS) for non-vaccinees [15]. C-statistics were calculated to ascertain the validity of PSs. When stratified by age group, actual age was not considered in calculating PSs or OR_DR._

In addition to the DR method, Cox hazard regression analysis was also conducted as a sensitivity analysis, using the time-dependent covariates of current year vaccination and preceding onset of influenza among siblings. Some studies investigating the effects of influenza vaccine on elderly mortality using electronic health record databases have suggested a potential bias towards apparent greater effectiveness [16–18]. The presence of this bias was examined with a Cox hazard model by investigating vaccine effectiveness during the 2013 pre-epidemic period, with minimum influenza circulation between October 1st and December 16th, the latter date being the day when the National Statistics announced the beginning of the epidemic season [19].

All statistical analyses were conducted using SPSS version 25.0 (IBM Corp., Armonk, NY) or SAS version 9.4 (SAS Institute Inc., Cary, NC). Tests were 2-tailed and had a significance level of 0.05.

## Results

**Fig. 1:**
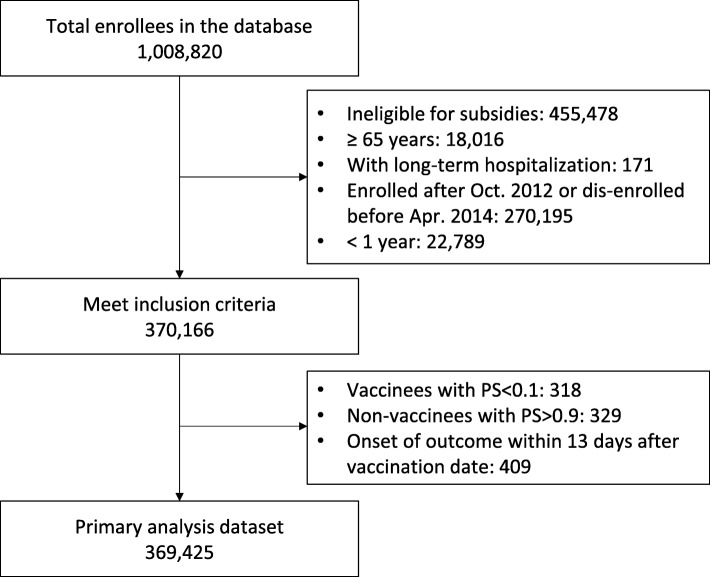
Subject disposition. PS: Propensity Score

**Fig. 2:**
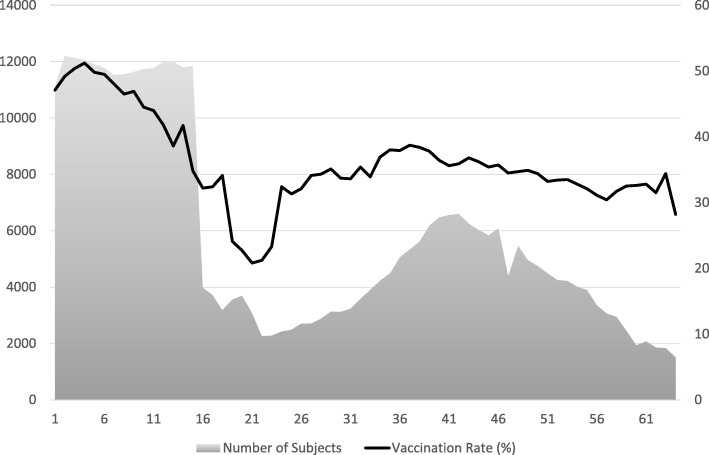
Number of subjects and annual vaccination rate by age in Japanese

Of a total of 1,008,820 people entered in the study database, 638,654 subjects did not meet the inclusion criteria for effectiveness analyses (Fig. 1). After further excluding 741 subjects with extreme PSs or with the onset of an outcome within 13 days after their vaccination date, 369,425 subjects were eligible for the primary analysis. Approximately 50% of all subjects were children aged under 16 years. Vaccination rate was 39.5% in subjects aged 1 to 64 years in the 2013/2014 season, while a low rate was particularly observed in subjects aged 19 to 23 years (Fig. 2).

In every age group, vaccinees and non-vaccinees significantly differed in almost all characteristics (Table 1).

**Table 1:** Characteristics of Japanese subjects in the primary analysis

Characteristics by age group	Vacnee	Non-vacnee	*P* Value ^a^
1–64 years			
N	145,784	223,641	
Age (median [interquartile range])	14 [7, 39]	22 [10, 43]	< 0.001 ^†^
Female	64,914 (44.5%)	98,589 (44.1%)	0.008 ^*^
Vacnation in the 2012/2013 season	91,541 (62.8%)	20,050 (9.0%)	< 0.001 ^*^
Insured persons (vs dependents) ^b^	45,859 (31.5%)	79,394 (35.5%)	< 0.001 ^*^
Number of children aged 0 to 15 years in a family (median [interquartile range]) ^c^	1 [0, 1]	1 [0, 1]	< 0.001 ^†^
Before influenza season			
Number of outpatient visits			
During office hours (median [range])	2 [0–118]	1 [0–112]	< 0.001 ^†^
Outside office hours (median [range])	0 [0–57]	0 [0–61]	< 0.001 ^†^
Emergency hospitalization	151 (0.1%)	348 (0.2%)	< 0.001 ^*^
High-risk condition	34,792 (23.9%)	43,735 (19.6%)	< 0.001 ^*^
During influenza season			
Preceding onset of influenza in family members	27,817 (19.1%)	38,620 (17.3%)	< 0.001 ^*^
1–4 years			
N	23,534	24,101	
Age (median [interquartile range])	3 [2, 4]	2 [2, 3]	< 0.001 ^†^
Female	11,387 (48.4%)	11,809 (49.0%)	0.184 ^*^
Vacnation in the 2012/2013 season	14,561 (61.9%)	2620 (10.9%)	< 0.001 ^*^
Insured persons (vs dependents) ^b^	–	–	–
Number of children aged 0 to 15 years in a family (median [interquartile range]) ^c^	1 [1, 1]	1 [1, 1]	0.005 ^†^
Before influenza season			
Number of outpatient visits			
During office hours (median [range])	5 [0–92]	4 [0–65]	< 0.001 ^†^
Outside office hours (median [range])	0 [0–19]	0 [0–16]	< 0.001 ^†^
Emergency hospitalization	15 (0.1%)	25 (0.1%)	0.155 ^*^
High-risk condition	7335 (31.2%)	7451 (30.9%)	0.552 ^*^
During influenza season			
Preceding onset of influenza in family members	5095 (21.6%)	5452 (22.6%)	0.011 ^*^
5–12 years			
N	43,535	50,247	
Age (median [interquartile range])	8 [6, 10]	9 [7, 11]	< 0.001 ^†^
Female	21,215 (48.7%)	24,227 (48.2%)	0.116 ^*^
Vacnation in the 2012/2013 season	33,310 (76.5%)	6118 (12.2%)	< 0.001 ^*^
Insured persons (vs dependents) ^b^	–	–	–
Number of children aged 0 to 15 years in a family (median [interquartile range]) ^c^	1 [1, 2]	1 [1, 2]	< 0.001 ^†^
Before influenza season			
Number of outpatient visits			
During office hours (median [range])	3 [0–85]	2 [0–73]	< 0.001 ^†^
Outside office hours (median [range])	0 [0–22]	0 [0–53]	< 0.001 ^†^
Emergency hospitalization	24 (0.1%)	25 (0.05%)	0.776 ^*^
High-risk condition	10,193 (23.4%)	9589 (19.1%)	< 0.001 ^*^
During influenza season			
Preceding onset of influenza in family members	10,424 (23.9%)	13,024 (25.9%)	< 0.001 ^*^
13–19 years			
N	18,123	31,993	
Age (median [interquartile range])	14 [13, 15]	15 [14, 16]	< 0.001 ^†^
Female	8811 (48.6%)	15,536 (48.6%)	0.904 ^*^
Vacnation in the 2012/2013 season	11,162 (61.6%)	4862 (15.2%)	< 0.001 ^*^
Insured persons (vs dependents) ^b^	158 (0.9%)	327 (1.0%)	0.107 ^*^
Number of children aged 0 to 15 years in a family (median [interquartile range]) ^c^	1 [0, 1]	1 [0, 1]	< 0.001 ^†^
Before influenza season			
Number of outpatient visits			
During office hours (median [range])	1 [0–74]	1 [0–61]	< 0.001 ^†^
Outside office hours (median [range])	0 [0–20]	0 [0–21]	< 0.001 ^†^
Emergency hospitalization	15 (0.1%)	47 (0.1%)	0.066 ^*^
High-risk condition	2219 (12.2%)	3044 (9.5%)	< 0.001 ^*^
During influenza season			
Preceding onset of influenza in family members	3479 (19.2%)	6094 (19.0%)	0.688 ^*^
20–64 years			
N	60,591	117,019	
Age (median [interquartile range])	42 [35, 50]	42 [34, 50]	< 0.001 ^†^
Female	23,501 (38.8%)	47,013 (40.2%)	< 0.001 ^*^
Vacnation in the 2012/2013 season	32,508 (53.7%)	6168 (5.3%)	< 0.001 ^*^
Insured persons (vs dependents) ^b^	45,701 (75.4%)	78,797 (67.3%)	< 0.001 ^*^
Number of children aged 0 to 15 years in a family (median [interquartile range]) ^c^	0 [0, 2]	0 [0, 1]	< 0.001 ^†^
Before influenza season			
Number of outpatient visits			
During office hours (median [range])	1 [0–118]	1 [0–112]	< 0.001 ^†^
Outside office hours (median [range])	0 [0–57]	0 [0–76]	< 0.001 ^†^
Emergency hospitalization	96 (0.2%)	250 (0.2%)	0.013 ^*^
High-risk condition	15,044 (24.8%)	23,525 (20.1%)	< 0.001 ^*^
During influenza season			
Preceding onset of influenza in family members	8819 (14.6%)	13,929 (11.9%)	< 0.001 ^*^

Subjects were stratified by age-group and vacnation status in the 2013/2014 influenza season

^a^The χ^2^ test was used for categorical variables (*) and the Mann-Whitney test for continuous variables (†) between vacnees and non-vacnees in the 2013/2014 influenza season

^b^All children aged under 18 years were treated as dependents

^c^Number of other dependents aged 0 to 15 years covered by the same insurance number

### Vaccine effectiveness for influenza onset

Current-year vaccination was associated with a significant reduction in the risk of influenza onset after adjusting for covariates in conventional logistic regression, with an OR of 0.756 (95% confidence interval (CI): 0.738–0.776) for influenza onset (Table 2). Female sex significantly reduced the OR (0.960, 95%CI: 0.940–0.980). In contrast, vaccination in the preceding season (1.051, 95%CI: 1.024–1.079), high-risk condition (1.138, 95%CI: 1.110–1.167), number of outpatient visits during office hours (1.016, 95%CI: 1.014–1.018), number of other dependents aged 0 to 15 years with the same insurance number (1.114, 95%CI: 1.100–1.128) and preceding onset of influenza in family members (1.263, 95%CI: 1.234–1.294) significantly increased the risk of influenza.

**Table 2:** Odds ratios (OR) of influenza onset versus vaccination and other characteristics in Japanese subjects: multivariate logistic regression

Variable	OR (95% CI)	*P* value
2013/2014 season vaccination	0.756 (0.738–0.776)	< 0.001
2012/2013 season vaccination	1.051 (1.024–1.079)	< 0.001
Insured persons (vs. dependents)	1.009 (0.965–1.056)	0.685
Female (vs. Male)	0.960 (0.940–0.980)	< 0.001
Number of children aged 0 to 15 years in a family ^a^	1.114 (1.100–1.128)	< 0.001
Before influenza season		
High-risk condition	1.138 (1.110–1.167)	< 0.001
Number of outpatient visits		
During office hours (per visit)	1.016 (1.014–1.018)	< 0.001
Outside office hours (per visit)	1.000 (0.987–1.013)	0.980
Emergency hospitalization	0.897 (0.658–1.224)	0.493
During influenza season		
Preceding onset of influenza in family members	1.263 (1.234–1.294)	< 0.001

Influenza onset was classified using the ICD-10 J10, J11 codes for clinical influenza. Age effects were modeled using linear tail-restricted cubic spline functions with 5 knots based on percentiles

^a^ Number of other dependents aged 0 to 15 years covered by the same insurance number

Given that influenza was defined based on ICD-10 codes only, the OR_DR_ was 0.775 (95%CI: 0.757–0.794) after doubly robust adjustment (Table 3). Sensitivity analysis using the outcome definition of J101 plus use of a rapid-testing kit gave similar OR_DR_ (0.793, 95%CI: 0.769–0.817).

**Table 3:** Incidence of influenza onset and odds ratios (OR_DR_) for influenza vaccination

Age	1–64 years	1–4 years	5–12 years	13–19 years	20–64 years
	No. (%)	No. (%)	No. (%)	No. (%)	No. (%)
Outcome definition	OR_DR_ (95% CI)	OR_DR_ (95% CI)	OR_DR_ (95% CI)	OR_DR_ (95% CI)	OR_DR_ (95% CI)
Influenza diagnosis codes only ^a^	50,487 (13.7)	9093 (19.1)	24,315 (25.9)	6366 (12.7)	10,674 (6.0)
	0.775 (0.757–0.794)	0.600 (0.567–0.633)	0.770 (0.741–0.800)	0.938 (0.879–0.997)	0.747 (0.708–0.786)
Influenza diagnosis codes + Prescription of antiviral drugs	46,425 (12.6)	7489 (15.7)	22,941 (24.5)	5975 (11.9)	9965 (5.6)
	0.772 (0.753–0.791)	0.562 (0.528–0.595)	0.775 (0.745–0.805)	0.941 (0.881–1.002)	0.744 (0.704–0.783)
Influenza diagnosis codes + Use of rapid testing	46,389 (12.6)	6528 (13.7)	23,539 (25.1)	6141 (12.3)	10,122 (5.7)
	0.784 (0.765–0.803)	0.572 (0.536–0.609)	0.773 (0.743–0.803)	0.930 (0.871–0.989)	0.750 (0.710–0.790)
ICD10 J101 code + Use of rapid testing ^b^	28,407 (7.7)	4052 (8.5)	14,594 (15.5)	3664 (7.3)	6061 (3.4)
	0.793 (0.769–0.817)	0.581 (0.536–0.626)	0.796 (0.760–0.833)	0.908 (0.835–0.981)	0.771 (0.719–0.824)

For each outcome, we excluded subjects who had the outcome within 13 days after vaccination. Therefore, the number of study subjects in an age strata differed by outcome definition. All confounders were adjusted using a doubly robust method

^a^ Defined by ICD-10 influenza diagnosis codes (J101, J110, J111, J118)

^b^ Defined by combination of ICD-10 influenza diagnosis codes (J101: influenza due to identified seasonal influenza virus) with use of a rapid-testing as outcome

The age-stratified OR_DR_s for influenza onset outcomes were all significantly below one, and were lowest in younger children aged 1 to 4 years (0.600, 95%CI: 0.567–0.633) and highest in older children aged 13 to 19 years (0.938, 95%CI: 0.879–0.997).

The C-statistics of the PSs for influenza vaccination were 0.766 or more in whole subjects and 0.709 or more in the age-stratified groups.

In most age groups, except for children aged 13 to 19 years, Cox hazard regression also produced significantly reduced hazard ratios with similar but slightly smaller vaccine effectiveness overall (Appendix 1) as compared with the ORs derived from logistic regression (Table 3). No significant, positive vaccine efficacy was seen during the pre-epidemic period in any age group (Appendix 2).

### Vaccine effectiveness for influenza complications

Significantly reduced OR_DR_s for all ages inclusive were observed for all secondary outcomes, ranging from 0.403 (emergency hospitalization with influenza or pneumonia) to 0.709 (hospitalization with influenza) (Table 4). In age group-stratified analyses, all OR_DR_s except for hospitalization with influenza and emergency hospitalization with influenza or pneumonia were significantly reduced. The OR_DR_ of hospitalization with influenza was significant only in the 1–4 years age group (0.529).

**Table 4:** Incidence of influenza complications and odds ratios (OR_DR_) for influenza vaccination

Age	1–64 years	1–4 years	5–12 years	13–19 years	20–64 years
	No. (%)	No. (%)	No. (%)	No. (%)	No. (%)
Outcome	OR_DR_ (95% CI)	OR_DR_ (95% CI)	OR_DR_ (95% CI)	OR_DR_ (95% CI)	OR_DR_ (95% CI)
Hospitalization with influenza ^a^	149 (0.04)	76 (0.2)	51 (0.1)	4 (0.01)	20 (0.01)
	0.709 (0.429–0.988)	0.529 (0.237–0.821)	0.724 (0.199–1.248)	5.547 (−6.851–17.945)	1.153 (−0.040–2.346)
Pneumonia ^b^	5025 (1.4)	2492 (5.2)	1354 (1.4)	197 (0.4)	990 (0.6)
	0.437 (0.405–0.469)	0.439 (0.396–0.483)	0.368 (0.313–0.422)	0.531 (0.340–0.722)	0.413 (0.337–0.489)
Respiratory tract diseases ^c^	180,177 (50.1)	40,303 (89.5)	62,499 (69.2)	21,269 (43.0)	57,240 (32.5)
	0.612 (0.603–0.621)	0.436 (0.407–0.464)	0.449 (0.434–0.464)	0.506 (0.483–0.529)	0.607 (0.591–0.623)
Hospitalization with respiratory tract diseases ^d^	1594 (0.4)	952 (2.0)	308 (0.3)	55 (0.1)	290 (0.2)
	0.441 (0.386–0.497)	0.491 (0.415–0.567)	0.303 (0.211–0.394)	0.314 (0.062–0.566)	0.356 (0.232–0.479)
Emergency hospitalization with influenza or pneumonia ^e^	59 (0.02)	10 (0.02)	9 (0.01)	5 (0.01)	35 (0.02)
	0.403 (0.126–0.680)	0.935 (−0.340–2.210)	0.122 (−0.083–0.328)	0.020 (− 0.009–0.048)	0.611 (0.062–1.160)

For each outcome, we excluded subjects who had the outcome within 13 days after vaccination. The number of study subjects in an age strata differed by outcome because vaccinees experiencing an outcome before vaccination were censored at the outcome onset and classified as non-vaccinees. All confounders were adjusted using a doubly robust method. Preceding onset of influenza among family members was included only for the analysis of “hospitalization with influenza” outcome

^a^ Hospitalization started within 3 days before or after the data of influenza diagnosis codes

^b^ Pneumonia including the ICD-10 codes J12-J18

^c^ Respiratory tract disease including the ICD-10 codes J00-J22, except for influenza diagnosis codes, J10 and J11

^d^ Hospitalization started within 7 days after diagnosis of respiratory tract diseases other than influenza

^e^ Emergency hospitalization with diagnoses of influenza or pneumonia

## Discussion

We conducted a large-scale effectiveness study of influenza vaccination in Japan using a health insurance claims database. Our study demonstrated that the effectiveness of influenza vaccination in preventing influenza onset was consistent across ages from 1 to 64 years old in the 2013/2014 season. Further, the risk of influenza complications, such as hospitalization with influenza, was significantly reduced in a real world setting, as previously reported [20, 21].

The incidence and OR_DR_s estimated in the present study likely reflect those for influenza-like illness, when the less stringent criterion of ICD10 codes only was applied. In contrast, the strictest criteria, namely the J101 code in combination with a rapid-testing kit, may provide the most conservative estimates of incidence and OR_DR_, albeit that this approach may lead to an increased number of false-negative cases [22]. However, given that a sensitivity analysis using a different definition of influenza onset and a different analysis technique with a Cox hazard model yielded similar risk estimates, the study results of influenza prevention appear to be robust. In calculating OR_DR_s for whole study subjects, the higher values of C-statistics for PSs using cubic spline function modeling of age-effect indicates better modeling than when age was treated as a linear effect (data not shown). Furthermore, given the high values of the C-statistics, the estimated PSs were considered to be valid.

The strongest effectiveness for the onset of influenza was observed in the vaccinated children aged 1 to 4 years. We speculate that this is probably due to careful, health-conscious behaviors of their families and high vaccination coverage. The US CDC recommends influenza vaccination for people at high risk of developing flu-related complications, including children aged under 5 years [12]. The Japanese Ministry of Health, Labour and Welfare recommends that, to decrease a child’s risk of influenza virus exposure, all family members remain apart from crowds, wash their hands after going outside and undergo vaccination as well [23]. In households with children, in general, family members are more likely to adopt health-conscious behaviors such as remaining apart from crowds and having vaccination every year for all family members, which may have resulted in greater effectiveness in aggregate [24]. The lowest effectiveness, observed in subjects aged 13 to 19 years, might be ascribable to lower influenza incidence and low immunization rates in this age group. Furthermore, because most of them are students and spend time with their peer group in school, they may have a higher chance of repeated exposure to various influenza viruses. However, significant increases in the OR estimates due to the number of siblings aged 0 to 15 years and preceding onset of influenza among family members may emphasize the importance of vaccination in household members; vaccine should be recommended regardless of age group.

Several risk factors for the onset of influenza were detected, including the number of children aged 0 to 15 years in a family. The possibility of introduction of influenza virus into a household would increase as the number of children increases [25]. Although employee insurance numbers helped identify familial infection, the possibility cannot be excluded that the dependents lived separately from the family. The present and our companion pediatric studies are the first to consider the risk of preceding onset of influenza in family members [5].

Influenza vaccination significantly reduced the risks of all outcomes of influenza complications in the 2013/2014 influenza season in the whole age population. Although some statistically insignificant OR_DR_s were observed in several age groups because of a small sample size and low incidences following age group stratification, vaccination effectively prevented most influenza complication events in most age groups.

Several cohort studies that used large-scale claims databases without laboratory-confirmed outcomes reported substantial bias in estimating vaccine effectiveness for elderly mortality [16, 18, 26]. Our study aimed to estimate the OR_DR_ for the incidence of medically attended influenza onset and complications in Japanese subjects aged less than 65 years, and therefore differed from these elderly mortality studies in terms of both study subject and endpoint. The use of mortality as the primary endpoint in elderly subjects may generate immortal-time bias given that the endpoint, mortality, affects the status of vaccine exposure [27]. That is, the elderly vaccinees cannot have died to bide time until they got vaccinated (immortal time) and would consequently be expected to have reduced mortality compared with non-vaccinees. The HR estimates for influenza onset and pneumonia during the pre-epidemic period in this study, together with the results of our pediatric companion study (Appendix 2), clearly demonstrated the absence of such bias [5].

The limitations and strengths of the present study are in essence identical to those described for our pediatric study [5]. Since we used the outcome definitions based on diagnostic codes recorded in the claims reimbursement, which are likely to be less specific than PCR-confirmed influenza and may represent medically attended influenza-like illness, our estimates may be biased toward null, together with the study design [5, 28]. Confounding by unmeasured variables may have been present due to the observational nature of the study. We used application for an influenza vaccination subsidy as a substitute for vaccination records, possibly leading to the misclassification of vaccination status. In addition, the lack of information on the number of vaccinations each pediatric subject received prevented us from considering variation in the number of vaccinations in assessing vaccine effectiveness in a season. Moreover, ORs were potentially over- or under-estimated due to the exclusion of subjects who had an outcome within 13 days post-vaccination. Although patients who met the definition for prolonged hospitalization were excluded from analyses, some patients with repeated short-term hospitalization could not be excluded. The accuracy of the reason given for hospitalization in the claims databases was not verifiable, potentially leading to the misclassification of events requiring hospitalization. The present study population consisted of employees and their dependents enrolled in healthcare insurance plans operated by private, blue chip companies. The covered workers were therefore likely to be socially advantaged, and the healthy worker effect might accordingly have been prominent. This would in turn result in a better health condition and biased estimations [29]. Additionally, children aged under 15 years old accounted for over 40% of our study population, which is higher than in the general population, possibly leading to overestimated ORs [30]. Finally, the risk of secondary infection in schools or office areas was not considered. In contrast, the DR likely conferred robustness on the estimated OR_DR_s, together with the use of a large-scale claims database, since it seems to be the methodology best suited to addressing channeling bias regarding being vaccinated, such as health-conscious behaviors [24].

## Conclusions

Our analysis confirmed that influenza vaccination in a large population of people aged 1 to 64 years significantly prevented the onset of influenza, and was similarly or more effective in reducing secondary risks due to influenza complications such as pneumonia in the 2013/2014 season. Since the study duration was limited to a single influenza season, annual study of each influenza season is required.

## Data Availability

All the data used in this study was provided by JMDC under the contract for the sole purpose of this study and not available for data sharing. Sharing the JMDC data set online is difficult owing to contractual agreements with JMDC. For inquiries about access to the data set used in this study, please contact JMDC (website, https://www.jmdc.co.jp).

## Appendix 1

**Table 5:** Vaccination and hazard ratios (HR) of influenza onset defined with ICD-10 codes only in Japanese subjects: time-dependent Cox hazard model

	Age							
	1–4 years (*n* = 47,732)		5–12 years (*n* = 93,946)		13–19 years (*n* = 50,119)		20–64 years (*n* = 177,960)	
Variable	HR (95% CI)	*P* value	HR (95% CI)	*P* value	HR (95% CI)	*P* value	HR (95% CI)	*P* value
2013/2014 season vaccination	0.705 (0.670–0.741)	< 0.001	0.878 (0.849–0.908)	< 0.001	1.022 (0.963–1.085)	0.468	0.855 (0.814–0.899)	< 0.001
2012/2013 season vaccination	0.822 (0.779–0.868)	< 0.001	1.008 (0.975–1.042)	0.646	1.076 (1.013–1.142)	0.017	0.986 (0.932–1.042)	0.616
Age	1.223 (1.199–1.247)	< 0.001	0.936 (0.931–0.942)	< 0.001	0.775 (0.762–0.789)	< 0.001	0.985 (0.983–0.987)	< 0.001
Insured persons (vs Dependents)	–	–	–	–	1.146 (0.747–1.756)	0.533	1.356 (1.277–1.439)	< 0.001
Female (vs. Male)	0.931 (0.894–0.971)	0.001	0.984 (0.960–1.009)	0.220	0.820 (0.780–0.861)	< 0.001	1.232 (1.166–1.302)	< 0.001
Number of children aged 0 to 15 years in a family ^a^	1.063 (1.035–1.092)	< 0.001	0.955 (0.938–0.971)	< 0.001	1.040 (1.007–1.074)	0.018	1.130 (1.107–1.153)	< 0.001
Before influenza season								
High-risk condition	1.111 (1.062–1.163)	< 0.001	1.085 (1.051–1.120)	< 0.001	1.102 (1.021–1.190)	0.013	1.139 (1.084–1.198)	< 0.001
Number of outpatient visits								
During office hours (per visit)	1.014 (1.010–1.018)	< 0.001	1.007 (1.004–1.010)	< 0.001	1.019 (1.011–1.027)	< 0.001	1.016 (1.011–1.020)	< 0.001
Outside office hours (per visit)	1.021 (0.995–1.048)	0.122	0.992 (0.974–1.011)	0.394	0.980 (0.948–1.014)	0.243	0.998 (0.978–1.019)	0.868
Emergency hospitalization	1.330 (0.715–2.475)	0.367	0.504 (0.252–1.008)	0.053	1.630 (0.924–2.876)	0.092	0.812 (0.504–1.308)	0.392
During influenza season								
Preceding onset of influenza in family members	4.671 (4.458–4.893)	< 0.001	2.261 (2.193–2.330)	< 0.001	1.906 (1.786–2.034)	< 0.001	4.530 (4.298–4.776)	< 0.001

## Appendix 2

**Table 6:** Hazard ratios (HR) for onsets of influenza and pneumonia during pre-epidemic periods (2013 Oct 1~ Dec 16) in Japanese subjects: time-dependent Cox hazard model

Outcome definition	Age							
	1–4 years		5–12 years		13–19 years		20–64 years	
	No. (%) HR (95% CI)	*P* value	No. (%) HR (95% CI)	*P* value	No. (%) HR (95% CI)	*P* value	No. (%) HR (95% CI)	*P* value
Influenza diagnosis codes only ^a^	112 (0.235)	0.991	225 (0.239)	0.806	44 (0.088)	0.665	112 (0.063)	0.281
	1.003 (0.612–1.644)		0.954 (0.653–1.392)		0.760 (0.219–2.636)		1.389 (0.764–2.524)	
ICD10 J101 code + Use of rapid testing ^b^	28 (0.059)	0.782	76 (0.081)	0.757	17 (0.034)	0.459	38 (0.021)	0.551
	1.143 (0.443–2.948)		0.906 (0.485–1.692)		0.452 (0.055–3.698)		1.349 (0.505–3.602)	
Pneumonia ^c^	1094 (2.293)	0.004	579 (0.615)	0.001	84 (0.168)	0.420	425 (0.239)	0.760
	1.319 (1.091–1.595)		1.543 (1.182–2.013)		0.551 (0.129–2.348)		0.932 (0.594–1.464)	

^a^Defined by ICD-10 influenza diagnosis codes (J101, J110, J111, J118)

^b^Defined by combination of ICD-10 influenza diagnosis codes (J101: influenza due to identified seasonal influenza virus) with use of a rapid-testing as outcome

^c^Pneumonia including the ICD-10 codes J12-J18

## Abbreviations

DR: Doubly robust method
OR: Odds ratio
PS: Propensity score
RTD: Respiratory tract diseases
