# Correction to: Influenza vaccination effectiveness for people aged under 65 years in Japan, 2013/ 2014 season: application of a doubly robust method to a large-scale, real-world dataset

**DOI:** 10.1186/s12879-019-4286-7

**Published:** 2019-08-12

**Authors:** Natsumi Shibata, Shinya Kimura, Takahiro Hoshino, Hisashi Urushihara

**Affiliations:** 1 Department of Drug Development and Regulatory Science, Faculty of Pharmacy, Keio University, 1-5-30 Shibakoen, Minato-ku, Tokyo, 105-8512 Japan; 2 Japan Medical Data Center Co., Ltd, Sumitomo Shibadaimon Building, 12F, 2-5-5 Shibadaimon, Minato-ku, Tokyo, 105-0012 Japan; 3 Department of Economics, Faculty of Economics, Keio University, 2-15-45 Mita, Minato-ku, Tokyo, 108-8345 Japan

**Correction to: BMC Infect Dis**

**https://doi.org/10.1186/s12879-019-4186-x**

After publication of the original article [1], in Table 1, in the second and third column, “Vacnee” and “Non-vacnee” should be replaced with “Vaccinee” and “Non-vaccinee”.

The original article has been corrected.

The publisher apologies for the inconvenience.
